# Non-professional-help-seeking among young people with depression: a qualitative study

**DOI:** 10.1186/1471-244X-14-124

**Published:** 2014-04-28

**Authors:** Angel Martínez-Hernáez, Susan M DiGiacomo, Natàlia Carceller-Maicas, Martín Correa-Urquiza, María Antonia Martorell-Poveda

**Affiliations:** 1 Medical Anthropology Research Center, Universitat Rovira i Virgili, Avinguda de Catalunya, Tarragona 35 43002, Spain; 2 Department of Anthropology, Philosophy and Social Work, Universitat Rovira i Virgili, Avinguda de Catalunya, Tarragona 35 43002, Spain; 3 Department of Anthropology, University of Massachusetts at Amherst, Machmer Hall, Amherst, MA 01003, USA; 4 Department of Nursing, Universitat Rovira i Virgili, Avinguda de Catalunya, Tarragona 35 43002, Spain

## Abstract

**Background:**

Adolescents and young adults often suffer from depression, but tend to avoid seeking professional help. The aim of this study was to explore the reasons for non-professional-help-seeking in a sample of young adults resident in Catalonia with depressive symptoms through a qualitative study. In addition, the subjects were invited to offer their recommendations for making mental health care services more accessible.

**Methods:**

We recruited 105 young persons (17–21 years of age) who had participated in a national survey on adolescents. The sample was divided into thirds, with 37 who had a previous diagnosis of depression, 33 who had self-perceived emotional distress, and 35 controls. The participants were interviewed in depth about their reasons for avoiding professional mental health care services, and the interview results were analyzed using both qualitative and cultural domain techniques and corroborated through comparison with the results of three focus groups.

**Results:**

Participants’ reasons for avoidance varied both by gender and according to prior experience with health services. Male study participants and female controls mainly understood depressive symptoms as normal and therefore not requiring treatment. Female participants with self-perceived distress were more likely to cite problems of access to treatment and fear of speaking to an unknown person about their problems. Females with a diagnosis expressed lack of trust in the benefits of treatment and fear of the social consequences of help-seeking. In their recommendations for best practices, the study participants suggested educational initiatives, as well as changes in the organization of mental health care services.

**Conclusions:**

A better understanding of the views of young people and a greater effort to involve them as active participants is important for facilitating help-seeking in this age group, and for adapting mental health care services to adolescent users and their social context.

## Background

Adolescents and young adults often suffer from both clinical and subclinical forms of depression and anxiety [1]. It is estimated that the lifetime prevalence of mood disorders among young people between the ages of 13 and 18 years is 14.3% and 31.9% for anxiety disorders [2]. To this we should add subthreshold depression and anxiety affecting 32% and 29.2% of adolescents respectively [3], and poorly specified and difficult-to-quantify negative mood states and depressive-type emotional distress [2-4] that may, in some cases, develop into major depression. One well-known study found a higher incidence and prevalence of depression among young people between the ages of 18 and 29 years than in other age groups, and a cumulative prevalence of 25% by age 24 [5]. However, most young people with clinical or subclinical depression do not seek professional help [6-9].

Avoidance or under-utilization of health care services is a constant among the adult population with mental health problems as well. A comparative study carried out in six European countries (Belgium, France, Germany, Italy, the Netherlands and Spain) found that among those defined as having a need for mental health care, only 51.7% were using some type of professional health care services, and as little as 25.1% had been seen by a mental health professional in the previous 12 months [10]. Among adolescents and young adults, the figures are even lower. Several studies have found that in many high-income countries, only 18% to 34% of adolescents with diagnosable depressive disorders or anxiety disorders are treated by mental health care professionals or general practitioners (GPs), even when these services are provided free of charge through a national health care system [6,11].

The literature indicates that in this age group under-utilization or avoidance is associated with a high degree of self-reliance, a negative and distrustful perception of GPs and mental health professionals [6,12], a lesser degree of mental health literacy [11,13], skepticism concerning psychiatric treatment [13], fear of the stigma associated with mental illness, shame [14], difficulty in expressing emotion [15,16], and a preference for seeking alternative forms of help through lay strategies and reliance on one’s social network [11,17,18]. Some of these studies point out that reluctance to seek professional help is more frequent among young men [19-21] because in the dominant cultural model of masculinity there is less tolerance for vulnerability, emotional fragility is deemed unacceptable, and use of these resources is perceived as a loss of personal autonomy. Other structural factors contribute to this situation in both sexes as well: limited access to psychiatric services, the generation gap, lack of awareness and information about services, and economic hardship [13], mainly but not only in societies that lack universal health care, as an indirect consequence of social inequalities in health-seeking processes.

A recent review article [11] notes an absence of high-quality research in studies of avoidance of mental health services by young people. Our reading of the literature leads us also to observe that reasons for avoidance tend to be conceptualized, in both qualitative and quantitative studies, as a series of discrete barriers [22] rather than as an integrated complex of factors or help-seeking process [23] linked to social context and previous experience (or lack thereof) with health care services. This fragmented way of approaching barriers to access has limited our knowledge of lay models and the associations between the different obstacles perceived by young people. In addition, most studies are limited to a description of factors that obstruct or facilitate access to health services, without taking into consideration the modifications in mental health care services proposed by young people themselves.

The aim of this study was to explore, through a qualitative research design, the reasons for non-professional-help-seeking in a sample of young adults resident in Catalonia who had participated in a national survey on adolescents. The use of qualitative data analysis techniques together with cultural domain analysis made it possible to find associations between different barriers to access. This in turn allowed us to delineate young people’s explanatory models of emotional distress and map their semantic networks of professional and lay help-seeking corresponding to gender and prior experience of both emotional distress and help-seeking. In addition, we conducted three focus groups to discuss our research results and to involve the participants actively in designing recommendations for improving the accessibility of mental health care services for young people.

## Methods

### Design and sampling

We conducted 105 face-to-face in-depth interviews and three focus groups with adolescents and young adults. The participants were recruited from the Panel of Families and Childhood (Pànel de Famílies i Infància, or PFI), a four-wave longitudinal survey designed and carried out by the Institute of Childhood and Urban Life (Institut d’Infància i Món Urbà) in Barcelona. Initiated in 2006 with adolescents resident in Catalonia who were born between 1990 and 1993, the survey incorporated a new cohort every year until 2009. Information was collected on negative mood states using a self-administered scale of distress in waves 2 and 3, and on diagnoses of depression or anxiety (reported by parents) in waves 1 and 4. It also included information on consumption of psychoactive substances, sociability, and familial and educational variables [24].

The sub-sample was recruited from all over Catalonia, rural areas included, using the Propensity Matching Score technique in order to yield three groups of 50 participants each: one with depression or anxiety diagnosed by a health professional in the first or fourth wave of the PFI, as reported by the parents in response to a direct question; a second group with self-perceived depressive distress in the second and third wave but without a diagnosis of depression or anxiety; and a control group with neither self-perceived distress nor a psychiatric diagnosis. Sample attrition occurred in cases of change of residence, inability to contact the subject, or subjects who declined to be interviewed, and in the end 105 subjects were interviewed (37 with a diagnosis, 33 with self-perceived distress, and 35 controls). The sociodemographic characteristics of the missing subjects were not significantly different from those of the subjects interviewed. The number of cases was sufficient to meet the data saturation point in qualitative research.

The study was approved and monitored by the Spanish Ministry of Science and Innovation, and by the Fundació La Marató de TV3, a Catalan not-for-profit foundation that raises money for biomedical research and funded our project. The study procedures were approved by the ethics committee of the Fundació Congrés Català de Salut Mental, an interdisciplinary entity for the promotion of mental health, and carried out in accordance with the ethical standards established by the Helsinki Declaration of 1964. Each participant and one adult with parental responsibility provided written informed consent.

### Interviews

The participants’ narratives on depression and help-seeking processes were elicited through in-depth interviews. For this interview, we designed an initial sketch in which an imaginary 17-year-old, John or Mary (see Additional file 1: Appendix 1), showed signs of depression according to DSM-IV-TR criteria, but without offering possible reasons or causes. The interviewer did not use the word “depression” or any other diagnostic category. The interviews were carried out in Spanish or in Catalan, depending on the subject’s mother tongue. The character in the sketch was always of the same sex as the person interviewed.

After reading the sketch aloud, the interviewer proceeded to the questionnaire, which took approximately an hour and a half to complete. Informants were asked to explain what they thought was happening to John or Mary, and the interview focused progressively on the subject’s own experience. The questionnaire included items agreed upon by the research team with the advice of several mental health professionals in psychology, psychiatry, social work and nursing. The questionnaire focused on lay explanatory models [25,26] of depression, lay strategies for confronting adversity, and possible reasons for avoiding professional help. Additional instruments validated in Spain were also used. These included the MISS (Manheim Interview on Social Support) [27] and the BDI-II (Beck Depression Inventory) [28]. The 11 interviewers (including MC, NC and MAM), all of whom were researchers in medical anthropology and/or psychology, participated in two working sessions to unify criteria and coordinate the dynamics of fieldwork and interviews. Interviewing was carried out between March and October 2011 at the convenience of the participants, who were contacted by telephone. Finally, each interviewer wrote up a reflexive evaluation of every interview completed.

### Focus groups

We (AMH, NC, MCU, SMD) conducted three focus groups, each comprising four to eight previously interviewed young adults of both sexes representing all three subgroups (diagnosis, undiagnosed distress, and control). At each session the preliminary results of the interviews were presented in order to facilitate a comparative discussion of the reasons for non-help-seeking and to elicit recommendations for improving the accessibility of mental health services.

### Analysis

Interviews and focus group discussions were audio or video recorded and transcribed verbatim. The data were managed using ATLAS-Ti 6.5 software [29]. Following an initial analysis to identify the principal themes in the data obtained, we (AMH, NC, MAM and MCU) initiated an independent analysis and subsequently discussed our observations to create a coding framework in accordance with the principles of grounded theory [30] and the ethnographic method, including discovery of emic or native semantic networks. We agreed that the point of saturation had been reached and it was not necessary to include additional cases. Cultural domain analysis techniques using UCINET 6.454 [31] made it possible to identify not only reasons for avoidance of professional services, but clusters or associations between these reasons, by transforming some qualitative data into binary matrices (respondent/cause of non-professional-help-seeking). The results of the analysis were compared with those obtained from the three focus groups.

## Results

### Reasons for non-professional-help-seeking

The sociodemographic characteristics of the participants in each subgroup and the results of the BDI-II questionnaire are shown in Table 1. All of the young people with BDI scores in the severe range were from the subgroup of participants with a diagnosis of depression and/or anxiety.

**Table 1:** Socio-demographic characteristics of the sample

		**Participants with diagnosis**		**Participants with emotional distress**		**Control participants**		**Total**
**Sex**	Male	13	39.4%	9	27.3%	11	33.3%	33
	Female	24	33.3%	24	33.3%	24	33.3%	72
	Total	37	35.2%	33	31.4%	35	33.3%	105
**Age**	17 years	5	33.3%	3	20.0%	7	46.7%	15
	18 years	10	47.6%	6	28.6%	5	23.8%	21
	19 years	12	38.7%	10	32.3%	9	29.0%	31
	20 years	7	25.0%	9	32.1%	12	42.9%	28
	21 years or older	3	30.0%	5	50.0%	2	20.0%	10
	Average	19.3		19.7		19.6		19.5
	Standard deviation	0.877		1.163		1.165		1.075
**Family income level**	Under 18000 €	6	31.6%	5	26.3%	8	42.1%	19
	18001-36000 €	18	40.9%	13	29.5%	13	29.5%	44
	36001 € or more	9	42.9%	7	33.3%	5	23.8%	21
	Missing data	4	21.1%	7	36.8%	8	42.1%	19
	Under 18000 €	6	31.6%	5	26.3%	8	42.1%	19
**Family structure 2010**	Single-parent family	11	34.4%	7	21.9%	14	43.8%	32
	Two-parent or reconstituted family	17	30.9%	19	34.5%	19	34.5%	55
	No information	9	50.0%	7	38.9%	2	11.1%	18
**BDI score**	Minimal	20	27.0%	24	32.4%	30	40.5%	74
	Mild	8	53.3%	4	26.7%	3	20.0%	15
	Moderate	4	36.4%	5	45.5%	2	18.2%	11
	Severe	5	100.0%	0	0.0%	0	0.0%	5
**Number of friends at Miss**	Average	8.79		8.15		8.76		8.57
	N	34		33		34		101
	Standard deviation	5.432		4.515		3.782		4.590
**Number of relatives at Miss**	Average	5.15		4.24		4.88		4.76
	N	34		33		34		101
	Standard deviation	2.105		1.640		1.737		1.861

Note: Young people with a diagnosis had a probability (OR) of 3.279 (CI 95%: 1.368 -7.856 p = 0.008) of having a BDI score in the severe, moderate, or mild range.

**Figure 1:**
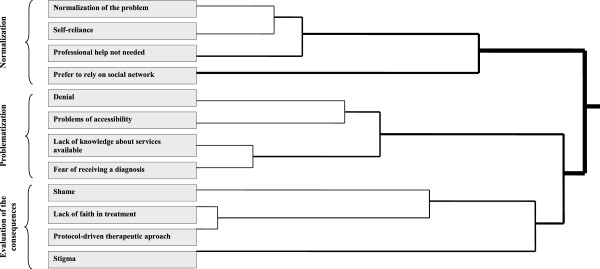
Cluster diagram of reasons for avoidance of professional help-seeking.

Of the 105 participants, only 21 (11 with a diagnosis, 7 with undiagnosed distress, and 3 from the control group) were clearly and explicitly inclined to seek professional help for the symptoms of depression. The common characteristic shared by these participants was a positive perception of or experience with mental health professionals. Nevertheless, they always included other resources, such as support from one’s social network or reliance on self, as alternatives to professional treatment. In the total sample, reasons for avoiding professional treatment can be summarized in 12 causes shown in Table 2. The most frequently cited reasons were “normalization of the problem”, “stigma”, “reliance on self”, and “no need for professional help”. By contrast, the least frequently cited reasons were “lack of accessibility”, “impersonal, protocol-driven treatment approach” and “no knowledge of available services”. These reasons should be understood as dynamic and interrelated in a semantic network that gives meaning to depressive emotional states and help-seeking. Figure 1 shows the clustering of reasons for avoidance, which fall into three large groups.

1. The first group consists of reasons associated with a condition described by participants as “being normally depressed”. In this case, the person’s own psychosocial resources (reliance on self and one’s social network) are thought to be sufficient to resolve problems of emotional distress.

2. The second group consists of reasons associated with the problematization of emotional distress and possible help-seeking. Here the obstacles to help-seeking lie in the subject (denial) and access to mental health services (not knowing how to access services, fear of speaking to a mental health professional and being diagnosed with a disorder, a user-unfriendly appointment system, inconvenient scheduling, or cost).

3. The third group consists of reasons associated with how participants evaluated the consequences, real or imagined, of professional help-seeking. These include social consequences such as stigma and shame, and the kind of treatment the subjects expected to receive from mental health services: lack of confidence in a positive outcome, and the expectation of a highly standardized, protocol-driven, diagnosis-oriented rather than patient-oriented treatment approach.

**Table 2:** Reasons for avoidance of professional help-seeking by subgroup

	**Female diagnosis**		**Female distress**		**Female control**		**Male diagnosis**		**Male distress**		**Male control**		**Total**
**Normalization of the problem**	4	12.9%	5	16.1%	11	35.5%	5	16.1%	2	6.5%	4	12.9%	31
**Stigma**	8	28.6%	4	14.3%	7	25.0%	3	10.7%	4	14.3%	2	7.1%	28
**Fear of receiving a diagnosis**	7	25.9%	8	29.6%	5	18.5%	2	7.4%	3	11.1%	2	7.4%	27
**Professional help not needed**	2	8.3%	2	8.3%	13	54.2%	2	8.3%	3	12.5%	2	8.3%	24
**Self-reliance**	4	16.7%	3	12.5%	4	16.7%	5	20.8%	4	16.7%	4	16.7%	24
**Prefer to rely on social network**	9	42.9%	0	0.0%	7	33.3%	3	14.3%	2	9.5%	0	0.0%	21
**Shame**	7	41.2%	2	11.8%	4	23.5%	1	5.9%	0	0.0%	3	17.6%	17
**Denial**	7	50.0%	1	7.1%	2	14.3%	1	7.1%	2	14.3%	1	7.1%	14
**Lack of faith in treatment**	6	42.9%	3	21.4%	2	14.3%	1	7.1%	1	7.1%	1	7.1%	14
**Problems of accessibility**	3	37.5%	2	25.0%	1	12.5%	0	0.0%	2	25.0%	0	0.0%	8
**Protocol-driven therapeutic approach**	3	42.9%	1	14.3%	1	14.3%	0	0.0%	1	14.3%	1	14.3%	7
**Lack of knowledge about services available**	0	0.0%	2	40.0%	1	20.0%	0	0.0%	2	40.0%	0	0.0%	5

What separates the first group from the second has to do with the lay definition of normal and pathological states. The boundary between the second and third groups corresponds to the decision of whether or not to seek professional help. Taken together, the three groups reveal a temporal logic in the form of an itinerary. Initially, young people regard their emotional distress as a normal experience. They rely on their own resources, which include their social networks, and consider professional help unnecessary. When their distress not only does not go away but becomes worse, they begin to view it as a problem and wonder whether they may have been in denial about its seriousness. At this point they begin to ask themselves what resources they should seek out, whether they would feel able to explain to someone else what is happening to them, and whether this would result in a diagnosis. Then they evaluate the consequences of help-seeking in social terms (shame and stigma) and in terms of whether they would receive care adequate to their needs. This model does not, however, occur in a fixed sequential order, but organizes the experience of young people in a dynamic and recursive way. For example, troubled young persons may foresee stigmatization as a consequence of help-seeking when they become aware of emotional distress as a problem, and subsequently attempt to normalize their feelings of depression.

### Normalizing emotional distress

Most of the young people in our sample defined the situation of the character in the sketch as a case of “depression”, but this should not necessarily be taken to mean that they are operating on the mental health professionals’ explanatory model of this concept. Their “depression” is a lay notion that, for the majority of the study participants, is a normal response to adversity that does not require psychotherapeutic or psychopharmacologic treatment. Being “normally depressed” is a consequence of everyday life situations: difficulty at school, problems with family or friends, even being in love, and for this reason solutions must be found in the context of everyday life and social relations (see Additional file 1: Appendix 2, point 2.1).

Participants believed that professional help-seeking should be reserved for situations that are more serious than the one described in the sketch. First, one should wait and see if the symptoms go away by themselves and take a proactive approach, going out with friends and participating in cathartic activities: listening to music, dancing, laughing, crying, and other ways of exorcising demons. The perception was that “depression” is more likely to go away when the depressed person faces up to it because he/she controls his/her own experience. On the contrary, help-seeking suggests loss of personal autonomy and control over one’s own life. One’s problems are one’s own, and should be resolved as such, because they are too personal to discuss with anyone else. Taking them to mental health services was seen as weakness of character that compromises their imagined future as independent and autonomous persons (see Additional file 1: Appendix 2, point 2.2).

Social networks, especially the family and one’s circle of friends, can help to mitigate emotional distress or find ways of resolving it, including by evaluating its severity and whether or not professional help is warranted (see Additional file 1: Appendix 2, point 2.3). There is, however, a gender difference: the young men were more likely to seek their friends’ help to “forget about” the problem, while young women wanted to “talk about” their problems.

For our male participants, “forgetting about” their troubles meant going out, having fun, partying. These activities were seen as necessary for taking the edge off emotional distress, not incompatible with “talking about” one’s problems but an activity that precedes it. Often “forgetting about” one’s troubles was a necessary first step that made it possible to “talk about” them later as a lay strategy for confronting emotional distress.

For young women, this strategy tended to work in reverse. Talking about their problems with friends allowed them to problematize and analyze what was wrong through negotiation with an interlocutor and begin to imagine a solution, preferably one their own social world was capable of generating. This was not incompatible with “forgetting about” their problems; they talked about them in order to be able to “forget about” them, inverting the order of their male counterparts’ strategies.

This interpretation is consistent with the observation that while the male participants had recourse to their social networks for normalizing their emotional distress (“forgetting about it”), the female participants tended to understand their social networks as a resource for problematizing it (“talking about it”), and therefore useful for producing awareness of emotional distress.

### Problematizing emotional distress

For our informants, their first awareness of the need for help-seeking began with getting beyond denial of the magnitude and severity of their emotional distress. This awareness emerged as a consequence of the duration of the distress or the appearance of unusual behavior, such as unwillingness to leave the house, loss of appetite, or crying for no apparent reason. The severity of the problem and therefore the need for professional help was a matter negotiated within the affected person’s social network of family and friends rather than an independent decision. The awareness of distress was the starting point of a process through which distress was constructed as a problem, which implied crossing the threshold from “normal” depression to pathology (see Additional file 1: Appendix 3, point 3.1). As other studies have also noted [22], among our informants the cutoff point for defining a situation as serious enough to require professional help was not as sharply defined as in the professional explanatory model, and was more likely to include emotional distress in the spectrum of normal responses to difficult life situations.

Denial was an obstacle to help-seeking cited principally by our female study participants with a diagnosis, and recognizing it, according to their narratives, involved “coming out of yourself”, removing the self-imposed blinders that “prevent you from seeing” the problem and “accepting what is happening.” At the same time, they feared the possibility of a diagnosis: “having something”, or that “they [mental health professionals] will find something.” Some informants with a diagnosis of depression told us that they realized that they had a mental health problem when they were unable to identify and explain the reason for their distress, and this made them all the more hesitant to speak with mental health professionals for fear they would be found to be in even worse shape than they suspected.

With self-awareness come the practical questions of how to initiate help-seeking (see Additional file 1: Appendix 3, point 3.2). Our study participants pointed to two obstacles that arose at this juncture: not knowing whom to consult, and problems of accessibility. In the first case, they noted difficulty in distinguishing between a psychiatrist and a psychologist, and how to know which was the better choice. In general, they opted for psychologists because they believed there was less stigma involved than in seeing a psychiatrist. Apart from a few exceptions, most associated psychiatrists only with pharmacologic treatment, which they believed was only necessary for persons suffering from something significantly worse than “normal depression”. Although some informants had difficulty specifying the reasons for their distress, most located both the causes and the solutions of their afflictions in the domain of everyday life and social relationships, not in the brain or the unconscious.

To explain their reluctance to seek professional help, our informants invoked the difficulty involved in getting an appointment to see someone through the public health care system, fear of forgetting the date of the appointment, referral delays, and the tension of waiting (“what are they going to tell me?”). They were unsure of how to go about locating a private therapist and found the cost prohibitive, and these obstacles, along with a certain lack of confidence in the benefits of therapy, contributed to non-help-seeking. Lay strategies re-emerged to take the place of professional help: spending hours alone in their rooms crying or trying to wait it out in the hope that “tomorrow is another day.”

### Evaluation of consequences

The stigma of mental illness was one of the reasons most cited in all three study groups for avoiding professional help. Stigma was understood in relational terms as a distortion of one’s social image and simultaneously as the impact of this distortion on one’s own self-image. In this way it operated on two levels – stigmatization by others (“they’ll think I’m crazy”), and self-stigmatization (“why can’t I get over this by myself?”) – and materialized as fear of rejection by family and friends, in the idea that one’s social prospects and job opportunities would be compromised, and in feelings of shame. Some informants even argued that help-seeking did not solve the problem of emotional distress, and so created not one problem but two: distress plus stigma. In their moral worlds, the stigma of being known as a person in treatment was a more powerful force than the emotional distress that impelled them to seek professional help. They felt vulnerably positioned, not only personally but also socially (“people will talk about you”). Stigma was probably the most universally cited reason across the three subgroups because it defines what is most at stake in the social worlds of young people: the loss of social ties and the possibility of exclusion or marginalization. Shame is a well-known form of social control of individual behavior through internalization of social rules. For the study participants, it was also a mechanism for containing emotional distress within the subjective realm, thereby avoiding the risk of rejection and the stigma associated with expressing distress in public social space, including mental health care services: “these things are so shameful that you can’t even talk about them” (see Additional file 1: Appendix 4, point 4.1).

Fears concerning the real or imagined consequences of mental health service use took the form of two principal reasons for avoidance (see Additional file 1: Appendix 4, point 4.2). The first was mistrust of mental health professionals both in terms of the efficacy of treatment and in terms of patient confidentiality; young people fear that what they say in private to a therapist may be communicated to their families. The second was a view of therapy as impersonal, protocol-driven and insensitive to the specificity of individual patients’ problems. As the participants saw it, mental health professionals tend to reduce particular and unique problems to standardized diagnoses and treatment approaches. Our participants explained that when they seek professional help for biographical and relational problems and receive a response framed in psychopathological terms they don’t understand, the resulting miscommunication leads to avoidance of mental health services.

### Reasons for avoidance by subgroup

**Figure 2:**
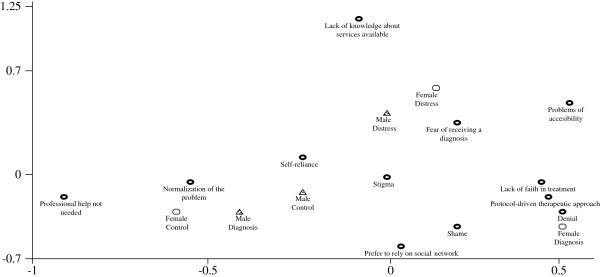
Correspondence analysis between sample subgroups and reasons for avoidance of professional care services.

The associations between the reasons described above and the three sample subgroups of young adults are shown in Figure 2:

1. The subgroups of male and female controls and male subjects with a diagnosis were associated with causes such as “normalization of the problem”, “professional help not necessary”, and “reliance on self.”

2. The group of female study participants with a diagnosis was located near “denial” as a reason for avoidance, although it was also close to “lack of trust in mental health professionals” and “impersonal treatment.”

3. Male and female participants with undiagnosed distress were associated with “fear of being diagnosed with a mental health problem”, “no knowledge of available services” and “lack of accessibility.”

Correspondence analysis reveals that the young women in the sample were located differently in the process of help-seeking or avoidance according to their previous experience with mental distress and mental health services use. The controls were more associated with normalization of emotional distress as a reason for avoidance. Participants with undiagnosed emotional distress were more associated with doubts about how and where to initiate treatment. Young women with a diagnosis were more associated with poor expectations concerning the therapeutic relationship. The young men were more clearly associated with an attitude of self-reliance, whether or not they had a diagnosis.

### Recommendations for improving accessibility

In the focus groups, the participants offered several recommendations for improving the accessibility of mental health care services which can be grouped into two large categories: normalizing help-seeking, and organization of services.

### Normalizing help-seeking

The participants indicated that a broadly based effort was necessary to eliminate or at least reduce the stigma associated with the use of mental health services in order to resituate recourse to them as a normal part of daily life. Schools were identified as a privileged space in which to carry out this normalization effort by challenging stereotyped notions about users of mental health services as “crazy” or “weak”. Among other measures, they believed that information on the diversity of users and potential users would help to dispel the notion that there is something strange or bizarre about requesting an appointment with a psychologist or psychiatrist. Educators were identified as people with an especially important role to play in this effort. The informants stressed the need for adolescents and young adults to have an active role in these processes of normalization in order to overcome the generation gap and the communication problems it entails. Their suggestions included workshops held in schools, not as occasional lectures by visiting mental health experts but with meaningful participation by adolescents and young adults, and mass-media exposure of the problems surrounding help-seeking in television series, news reports and advertising in a way that would allow young people themselves to speak openly about their experiences and provide information about talking with mental health professionals, where to find mental health services, and how to get access to them. What all these recommendations have in common is a clear preference for horizontal rather than vertical styles and forms of communication.

### Organization of mental health care services

Participants considered that in order to make help-seeking easier, the services provided should be less distant, more personalized, and have better continuity at different levels: between pediatricians and GPs, child and adult mental health services, and GPs and mental health professionals. They wanted to see more flexibility and less formality, and more attention to patients’ subjective concerns and less emphasis on diagnosis and protocol-driven treatment, which they considered to be an obstacle in itself to help-seeking; in sum, they wanted care, not services. Among other initiatives, they proposed that the first visit with a therapist take place at the patient’s home or in a neutral location (for example, while taking a walk) in order to improve accessibility and facilitate good patient-therapist communication. Delays in referral to mental health services can be as long as three months, and they recommended shortening them as much as possible and spacing subsequent visits a week apart rather than two or even four weeks. They noted the need for flexibility in adapting appointment schedules, since public mental health care services generally operate during school hours, and the need to justify their absence makes young patients feel conspicuous and vulnerable to stigma and shame. They recommended that therapists not spend all their time during the appointment taking notes.

## Discussion

This study explores young people’s reasons for avoiding professional help for depressive emotional states, and offers recommendations generated by the participants themselves for improving the accessibility of mental health services. Young people’s reasons for non-help-seeking are strongly conditioned by gender. The young women in our sample tended to explain their avoidance by reference to prior experience of distress and help-seeking, while the young men tended, independently of previous experience, to treat their perceived distress as a normal part of life. This finding is consistent with a lower propensity on the part of young men to seek professional help because of cultural notions of masculinity that stress self-reliance as a value. As other studies have also demonstrated [13,19-21], psychological symptoms and gender were shown to be more relevant predictors of help-seeking than personality characteristics [32]. Among our informants, a greater tendency toward avoidance among male participants was associated with a greater preference for “normalization” of symptoms, but also with strategies that delayed awareness and recognition of a problem. In fact, for our male participants, self-control preceded awareness of the problem.

By contrast, the preferred strategy among the young women in our sample was to mobilize their social networks, especially peers, to talk about what was troubling them as a way of bringing the sources of their distress to conscious awareness and problematizing their symptoms. For our female study participants, awareness of the problem preceded self-control.

The results of this study are partially consistent with those reported in a recent systematic review [11] that includes the most important studies carried out in Australia, the United States and the United Kingdom among other countries, and identifies the primary causes for avoiding professional services as stigma, lack of confidentiality and trust, difficulty identifying the symptoms of mental illness, concern about the characteristics of the provider, and reliance on self. In our study, the most frequently cited causes for avoidance were normalization of the problem, stigma, fear of receiving a diagnosis, professional help not needed, and self-reliance. The greater weight of the lay explanatory model of “being normally depressed” (normalization of the problem) in our study can be explained not only by the inclusion of controls, but also by the fact that most studies interpret this tendency toward normalization as a low level of mental health literacy among young people. From our point of view, this interpretation contributes little of relevance to an understanding of lay perspectives because it is medico-centric and does not take into consideration the lifeworld of young people and the meanings they ascribe to their experience. The idea of normalization allows us to get closer to their reality, and taking their perspective into account may be of value to mental health care professionals as they work to create a therapeutic alliance with their patients.

The process of help-seeking involves progressive transfer of subjective psychological problems to the social arena, which may involve a search for lay solutions (friends, social networks, and family), professional solutions, or both. Our data show that the choice of one resource over another does not depend on a decision made by an abstractly conceived rational actor who views access to health services as the solution to his or her problems, but on a process through which emotional distress is negotiated at the subjective level between individuals and their social networks. This is why the reasons for avoidance should not be seen as discrete “barriers”, but processually [33,34] or, more specifically, as a cycle of avoidance [22]. Some authors have indicated that the process begins with awareness of the problem, and develops subsequently with expression of symptoms and a need for support, availability of sources of help, and finally willingness to seek out and disclose the problem to these sources of help [14]. Our results suggest, however, that awareness of the problem seems to emerge from repeated attempts to normalize its symptoms. This is why our model proposes three phases (normalization of distress, problematization of distress and evaluation of consequences) of help-seeking that are derived from the tacit explanatory model of young people themselves (Figure 1). Its logic is not static but dynamic, since attempts at normalization may be simultaneous with or subsequent to awareness of the problem, especially among male study participants.

### Strengths and limitations

To date we have found no studies comparing the reasons for non-professional-help-seeking by gender and prior experience of emotional distress (diagnosed disorder, self-perceived emotional distress, and controls). Through a detailed qualitative analysis, this study contributes to our understanding of different subtypes of avoidance in a vulnerable population, going beyond a conceptualization of barriers to access as discrete phenomena in isolation from each other. This is also the first systematic study of non-professional-help-seeking among adolescents and young adults with depressive emotional distress to be carried out in Spain in general and in Catalonia in particular. In other European countries with a national health service structured along similar lines, our study furnishes a basis for comparative research.

The most evident limitations of the study have to do with the lack of triangulation with observational data on the use or avoidance of mental health services. A further limitation is the absence of the perspective of mental health professionals, although this article reports on results from the second phase of the study only. In the third phase, we organized focus groups of professionals and one mixed group including both young people and professionals with the purpose of creating a guidebook of best practices and a documentary video [35] to improve the accessibility of mental health services and tailor treatment more closely to the needs of this age group.

## Conclusions

Most severe and recurrent mental health problems appear before the age of 25, and those in the depressive spectrum have significant clinical and social implications that include suicide, poor social adjustment, chronicity, consumption of psychoactive substances, and severe mental disorders in adult life [36]. Preventive strategies are helpful, but neither these nor the organization of mental health services are always well suited to the needs of this population, as recent studies have pointed out [37-39]. This study contributes knowledge that furnishes a basis for policy modifications that would result in mental health services better adapted to young people’s needs. For example, our results show that promotion of mental health and strategies for increasing use of professional services among young adults need to be gender-specific, taking into account the dynamic we observed among young men, who first try to “forget about” their distress as a prelude to “talking about” it. It is not by chance that in their recommendations, our male study participants suggested talking with a therapist over coffee or while taking a walk.

The young people who participated in this project offered suggestions for greater accessibility that can and should be taken into consideration. As one of the study participants pointed out, if you are depressed you have not one problem but two: the disorder plus stigma. The normalization of mental health service use can be an antidote to the normalization of depression among young adults, and help to increase mental health literacy. If young people come to see these services as a resource for maintaining mental health and not only as a resource for treating mental disorders, users would be less likely to feel stigmatized, and this in turn would help to remove one of the barriers to access. The same may be said of the need for clinical approaches based on attaching importance to the patient’s biographical specificity, and on flexibility, at least in the first encounter between therapist and patient, in order to facilitate the development of a therapeutic alliance.

A final implication of our research is the utility of qualitative approaches in the study of non-help-seeking. To the extent that data collection is carried out dialogically through interviews and permits young people to express their problems and strategies for resolving them freely and in their own words, this encounter generates more than data: it creates social ties. In stimulating reflection by both study participants and researchers, it functions unobtrusively as a form of prevention and as an invitation to young people to engage with mental health care providers and contribute their insights toward improving the accessibility of services.

## Competing interests

All the authors declare that they have no competing interests.

## Authors’ contributions

AMH conceived and designed the study, analyzed and interpreted the data, and wrote the first drafts of this article. SMD designed the study, analyzed and interpreted the data, and rewrote the final version of this article with AMH. NCM, MCU and MAM participated in data collection, analyzed and interpreted the data, and reviewed the article’s intellectual content. All authors read and approved the final manuscript.

## Pre-publication history

The pre-publication history for this paper can be accessed here:

http://www.biomedcentral.com/1471-244X/14/124/prepub

## Supplementary Material

Additional file 1: Appendices **Appendix 1**: The qualitative questionnaire. **Appendix 2**: Normalization of the problem. Quotations to support and illustrate the key themes. **Appendix 3**: Problematization. Quotations to support and illustrate the key themes. **Appendix 4**: Evaluating consequences. Quotations to support and illustrate the key themes. Click here for file
