# Supplementary material for: Non-professional-help-seeking among young people with depression: a qualitative study
Source: BMC Psychiatry. 2014 Apr 28;14:124. doi: 10.1186/1471-244X-14-124 (PMC4021372; doi:10.1186/1471-244X-14-124)
Supplement: Additional file 1: Appendices — Appendix 1: The qualitative questionnaire. Appendix 2: Normalization of the problem. Quotations to support and illustrate the key themes. Appendix 3: Problematization. Quotations to support and illustrate the key themes. Appendix 4: Evaluating consequences. Quotations to support and illustrate the key themes. [file 1471-244X-14-124-S1.docx]

# Additional files

### Additional file 1 – Appendix 1: The qualitative questionnaire

Mary/John is 17 and has felt bad for the last two weeks. She/he feels sad and empty and sometimes irritable. She/he finds it very difficult to concentrate. She/he no longer finds pleasure in the things that she/he has always enjoyed and has lost her/his appetite. She/he finds it very difficult to sleep at night and has no energy during the day to carry out his/her normal activities.

1. What do you think is wrong with Mary/John?
2. Which different names are given to what is happening to her/him?
3. Can you tell me which words people your age use to talk about these states?
4. Have you ever found yourself in this situation or in a similar one? If you have, can you describe it to us? If not, do you know anyone your age who has been in this situation? If so, how do you remember that situation? How old were you?
5. What could be the causes of Mary/John’s condition?
6. Do you think girls and boys experience emotional distress for different reasons?
7. If you have ever felt this way, what caused it?
8. Do you think that emotional distress is experienced differently when you are 15 than when you are 18? Why?
9. How can you recognize when somebody, either a boy or a girl, is in the same situation as Mary/John (gestures, facial expression, attitude, etc.)?
10. Do you think the signs of emotional distress are different in boys and girls? How?
11. How do you know when you are feeling bad? What happens to you? How do you feel? What do you do? What do you stop doing? How well does this work for you?
12. What could Mary/John do to feel better? (list the possibilities)
13. How could she/he be helped? (list the possibilities)
14. If the respondent does not mention any health professional, say to him/her: Mary/John does not want to go to a health professional for help. Why do you think this is?
15. What professional health resources/people do you know of? (list them)
16. What do you think about each of these options?
17. Which ones would you recommend to Mary/John? Why?
18. Have any of your friends ever been to see any of these professionals? (If the answer is yes) How do you know? Did they tell you? How did it go for them? (well, badly, etc.); (If the answer is no) Why do you think they haven’t gone?
19. Would you go to one of these professionals if you felt like Mary/John? Why? What would you expect from these professionals?
20. If you decided not to go to one of these professionals, who would you go to for help (person, group/institution, etc.)? Why?
21. If you went to one of these professionals, would you go to more than one person/group/institution, etc.? Why?
22. Has any member of your family ever been to see one of these professionals? (if the answer is yes) How did it go for them? (if the answer is no) Why haven’t they gone?
23. What does your family think about these professional resources?
24. What would you do if you were Mary’s/John’s friend?
25. What could her/his family do to help her/him?
26. Which strategies do you think you use most often to deal with your problems?
27. Which strategies do your friends use?
28. Which strategies does your family use?
29. What would you like others to do to help you feel better?
30. Do you belong to any youth association, choral society, hiking group, church-affiliated group, dance group, art club, or other voluntary association?
31. If so, which one(s)? Why? What do you get out of it? How do you feel in these groups?
32. If not, why? Have you ever belonged to any group of this kind?
33. If so, which one(s)? When? For how long? Why did you join? How did you feel in the group? When did you leave? Why did you leave?
34. Do you have the same number of friends now as you had when you were 15? If the number of friends you have has changed, why do you think this is?
35. Do you think this has changed the way you think about friendship?
36. Do you tell your friends more things now than you used to? Why do you think this is?
37. Have the kinds of things you talk about with your friends changed? If so, why do you think this is?
38. Have the things that worry you changed since you were 15? If so, what did they used to be? What are they now? Why do you think this change has happened?

### Additional file 2 – Appendix 2: Normalization of the problem. Quotations to support and illustrate the key themes.

Words added by the authors to a quotation to improve the reader’s understanding are indicated in square brackets [ ]. Each quotation is followed by a summary of the characteristics of the specific participant, including participant number at the beginning; gender; subgroup; and Beck Depression Inventory (BDI-II) score.

2.1. “Being normally depressed”

Q: Would you see a therapist if you felt like Mary/John?

A: No, because it’s not a serious problem. It happens to me, and I don’t feel I need to go to a psychologist. It’s normal for that to happen sometimes. You’d go to a psychologist for something more serious, not for something silly like this just because you feel depressed. [157 female control, BDI-II Minimal (11)]

A: I don’t think she should to go to a therapist. Everybody’s got problems, all kinds of depressions and mini-depressions, and I don’t think you have to run to a specialist every time you have a problem. [154 female control, BDI-II Minimal (4)]

A: To go to a therapist you’d have to have a very serious problem, something you couldn’t handle in any other way. [142 female control, BDI-II Minimal (2)]

2.2. No need for professional help

Q: Would you go to a therapist if you felt like Mary/John?

A: Well, I admit that right now I’m not happy, but I sure don’t need a psychologist or a psychiatrist. I’d have to be in really bad shape. [179 male control, BDI-II Minimal (2)]

A: You don’t need an expert to tell you what’s wrong with you. If it’s more than you can handle alone…well, OK. Maybe I need somebody to listen to me, but they don’t need a degree in anything to give advice. [28 female with diagnosis, BDI-II Minimal (5)]

A: If you go to them for help, it makes you feel weak. You think, “shit, I’ll never be able to do anything in life by myself.” [148 female control, BDI-II Minimal (13)]

2.3. Reliance on social network

Q: Would you go to a therapist if you felt like Mary/John?

A: If you have friends who can help you, what do you need a therapist for?

[100 male with diagnosis, BDI-II Minimal (4)]

A: First she should go out with her friends, and if there’s someone she’s really close to, talk it over with her and see what she says, and if she still feels bad, then she should see a therapist. [17 female with diagnosis, BDI-II Minimal (7)]

### Additional file 3 – Appendix 3: Problematization. Quotations to support and illustrate the key themes.

Words added by the authors to a quotation to improve the reader’s understanding are indicated in square brackets [ ]. Each quotation is followed by a summary of the characteristics of the specific participant, including participant number at the beginning; gender; subgroup; and Beck Depression Inventory (BDI-II) score.

3.1. From normality to awareness of a problem

Q: Would you go to a therapist if you felt like Mary/John?

A: What’s happening [to Mary] isn’t serious enough to go to a psychologist. She can get over it on her own.

Q: When do you think that something is so seriously wrong that you should go to a psychologist?

A: When you start doing strange things. You spend the whole day in the house, doing nothing, you don’t even get out of bed. You stop eating. When you start acting really weird. [59 female with undiagnosed distress, BDI-II Minimal (8)]

Q: What do you think about professional health resources/people?

A: Going to a psychologist isn’t really necessary. In most cases they don’t help much. Well, I guess sometimes they do, but only in extreme cases. [114 male with undiagnosed distress, BDI-II Minimal (7)]

Q: Mary/John doesn’t want to see a therapist of any kind for help. Why do you think this is?

A: They don’t realize they have a problem. They think, “I feel bad now, but it’ll go away”, and they don’t see the consequences it might have. [1 female with diagnosis, BDI-II Minimal (12)]

A: So she won’t have to face herself. We’re afraid of ourselves. Afraid of what this person will tell us about ourselves, afraid of finding out why we feel so bad. [7 female with diagnosis, BDI-II Minimal (3)]

3.2. Lack of knowledge of available services and problems of accessibility

Q: Mary/John doesn’t want to see a therapist. Why do you think this is?

A: Because he doesn’t want to go through all that. First you have to go to the doctor, to get an appointment. You have to make a note of it so you won’t forget. Then you have to go there. Waiting, while you wonder what they’re going to tell you. [116 male with undiagnosed distress, BDI-II Minimal (9)]

A: If they weren’t so expensive, or you didn’t have to wait so long to see them, because there aren’t many in the public health care system, and you might have to wait two months between visits. I’m not about to do that. [47 female with undiagnosed distress, BDI-II Minimal (4)]

A: It’s not easy to explain how you feel, it’s really hard. And before she can see a therapist, she has to ask her mother first, because she’s still a minor. So you have to tell your mother that you have a problem, and she’ll want to know what it is… [169 female control, BDI-II Minimal (13)]

### Additional file 4 – Appendix 4: Evaluating consequences. Quotations to support and illustrate the key themes.

Words added by the authors to a quotation to improve the reader’s understanding are indicated in square brackets [ ]. Each quotation is followed by a summary of the characteristics of the specific participant, including participant number at the beginning; gender; subgroup; and Beck Depression Inventory (BDI-II) score.

4.1. Stigma and shame

Q: Mary/John does not want to see a therapist. Why do you think this is?

A: Fear of rejection. You need help, but your fear of being rejected is stronger than your desire to feel better. [169 female control, BDI-II Minimal (13)]

A: She probably doesn’t want to talk to anyone about what’s bothering her. She’s ashamed, she feels they’re going to analyze her, that they’ll laugh at her…

Q: She’s afraid that who would laugh at her? The therapist…?

A: Yes, she probably thinks these things are so shameful that you can’t even talk about them, they’ll probably think she’s crazy and tell her parents, and she doesn’t want that. [26 female with undiagnosed distress, BDI-II Severe (31)]

A: People will talk about you. When you break a leg it’s great when everybody sees you in a cast, but when you have to go to a psychologist it’s not so great for everybody to know about it. You don’t want them to know you have problems. [52 female with undiagnosed distress, BDI-II Minimal (5)]

A: My mother told me to go see the psychologist, but I said no, that’s for crazy people, and I’m not crazy. [2 female with diagnosis, BDI-II Mild (14)]

A: If you feel bad, you’re seeing a psychologist, and on top of that you think that other people think you’re crazy, well…it’s the worst of all possible worlds! [78 female with undiagnosed distress, BDI-II: Moderate (24)]

A: I was too embarrassed. I never said where I was going. Because I went during school hours, and I never said it. Instead I said, “I’m going to the doctor.” **[**95 male with diagnosis, BDI-II Minimal (2)]

4.2. Lack of trust in mental health professionals; impersonal treatment

Q: Mary/John does not want to go to a therapist. Why do you think this is?

A: Lots of people don’t trust them, they don’t think they can help them. If you don’t know them, how can they help you? They don’t know what’s inside your head. [186 male control, BDI-II Minimal (13)]

A: They don’t always help. And it’s very hard to open up to some stranger who’s going to look at you as if you were some kind of specimen. It’s more helpful to talk to someone who’s close to you, or maybe not but at least someone you know, than some guy in a white coat who just sits there writing down everything you say. [38 female with diagnosis, BDI-II Moderate (21)]

A: Psychologists have this checklist, and if you have this and this, then you’re…this. No. My problem isn’t the same as someone else’s problem. Why do you have to treat me this way if I’m not like this other person? She doesn’t feel the same things I do, we’re not alike. You can’t treat me the same as you treat the person who comes in after me. We’re very different. These are two parallel lives that don’t have anything to do with each other, you can’t treat me the same as her. [28 female with diagnosis, BDI-II Minimal (5)]

A: One of my friends went to a private psychologist, and just for that reason he didn’t trust him because he thought, “if my father is paying him, he’s going to go and tell him everything because he’s being paid to.” [76 female with undiagnosed distress, BDI-II Minimal (13)]
